# Transcriptome Analysis of Newt Lens Regeneration Reveals Distinct Gradients in Gene Expression Patterns

**DOI:** 10.1371/journal.pone.0061445

**Published:** 2013-04-16

**Authors:** Konstantinos Sousounis, Mario Looso, Nobuyasu Maki, Clifford J. Ivester, Thomas Braun, Panagiotis A. Tsonis

**Affiliations:** 1 Department of Biology and Center for Tissue Regeneration and Engineering at Dayton, University of Dayton, Dayton, Ohio, United States of America; 2 Department of Bioinformatics, Max-Planck-Institute for Heart and Lung Research, Bad Nauheim, Germany; 3 Department of Cardiac Development and Remodeling, Max-Planck-Institute for Heart and Lung Research, Bad Nauheim, Germany; Indiana University School of Medicine, United States of America

## Abstract

Regeneration of the lens in newts is quite a unique process. The lens is removed in its entirety and regeneration ensues from the pigment epithelial cells of the dorsal iris via transdifferentiation. The same type of cells from the ventral iris are not capable of regenerating a lens. It is, thus, expected that differences between dorsal and ventral iris during the process of regeneration might provide important clues pertaining to the mechanism of regeneration. In this paper, we employed next generation RNA-seq to determine gene expression patterns during lens regeneration in *Notophthalmus viridescens*. The expression of more than 38,000 transcripts was compared between dorsal and ventral iris. Although very few genes were found to be dorsal- or ventral-specific, certain groups of genes were up-regulated specifically in the dorsal iris. These genes are involved in cell cycle, gene regulation, cytoskeleton and immune response. In addition, the expression of six highly regulated genes, TBX5, FGF10, UNC5B, VAX2, NR2F5, and NTN1, was verified using qRT-PCR. These graded gene expression patterns provide insight into the mechanism of lens regeneration, the markers that are specific to dorsal or ventral iris, and layout a map for future studies in the field.

## Introduction

Amphibians, especially newts, possess regenerative capabilities that are missing in higher vertebrates. Newts can regenerate their limbs, brain, heart, tail with spinal cord and other tissues. Interestingly, newts can also regenerate the lens after its complete removal (lentectomy). This system provides many advantages for regenerative studies because the whole organ (lens) is being removed. Lens regeneration occurs from the iris by a process that involves the transdifferentiation of pigmented epithelial cells (PECs) to lens cells. Another interesting aspect of this process is that lens regeneration occurs only from the dorsal and never from the ventral iris. This allows the use of the ventral iris as a natural non-regenerative control in lens regeneration experiments [1], [2], [3].

Two major hallmarks of lens regeneration are the re-entry of the cell cycle 4 days post-lentectomy (dpl) and the formation of a dedifferentiated vesicle 8 dpl [4]. Interestingly, both dorsal and ventral iris cells re-enter the cell cycle [5]. In the past, limited expression studies, either using individual gene probes or small-scale microarray analysis have indicated that dorsal and ventral irises show no major differences in gene expression. In other words, most of the examined genes were expressed in both irises. Thus, to date no clear expression pattern has emerged to account for the ability of the dorsal iris to be the source of the regenerating lens. More recently a microarray analysis during early stages of lens regeneration was performed. In that study expression in 1, 3 and 5 dpl from the dorsal or the ventral iris was compared with the corresponding intact iris (0 day). While that study indicated regulation in genes related to DNA repair, extracellular matrix and redox homeostasis, direct comparisons between gene expression in dorsal and ventral iris could not be assessed. Thus, a direct comparison of transcriptomes was needed to delineate global gene expression differences between dorsal and ventral iris during lens regeneration in newts [6], [7].

Next-generation high-throughput techniques allow transcriptome analysis based on de novo assemblies, making them extremely useful for non-model systems like the newt. Here, we investigate transcriptional changes during newt lens regeneration in an attempt to identify patterns that provide clues for the ability of dorsal iris but not the ventral to transdifferentiate. We focused on 4 dpl and 8 dpl for both dorsal and ventral iris as these time points are crucial stages for lens regeneration because these time points encompass the events of cell cycle re-entry and dedifferentiation. For RNA-seq we used a de novo assembled trancriptome making use of short Illumina and longer 454 and Sanger reads [8].

Here we report and for the first time the expression of more than 38,000 annotated transcripts in dorsal and ventral iris during lens regeneration. The analysis has been focused on the quantitative and qualitative differences between dorsal and ventral iris of those transcripts. We found very few genes to be dorsal or ventral iris-specific. However, certain cohorts of genes grouped according to their function were found to be preferentially up-regulated in the dorsal iris. Genes involved in the cell cycle, transcriptional apparatus, cytoskeleton and immune response are among those with much higher expression in the dorsal than the ventral iris. This graded expression might provide robust regulation that allows the dorsal iris to “win” over the ventral iris.

## Methods

### Animals - Lentectomy

Handle and operations on *Notophthalmus viridescens* have been described previously [6]. Briefly, newts were purchased from Charles Sullivan Inc. Newt Farm. Newts were anesthetized in 0.1%(w/v) ethyl-3-aminobenzoate methanesulfonic acid (MS222; Sigma) in phosphate buffered saline. Lentectomy was performed using a scalpel to incise the cornea and tweezers to pull out the lens through the incision. For the present study newts were kept for 4 and 8 dpl before tissue harvest.

### Ethics Statement

All procedures involving animals were approved by the University of Dayton Institutional Animal Care and Use Committee (IACUC; Protocol ID: 011-02). All surgical procedures were performed in anesthetized with MS222 newts. All appropriate procedures were used in order to alleviate pain and distress while working with newts.

### Tissue Harvest and RNA Extraction for qRT-PCR

**Figure 1:**
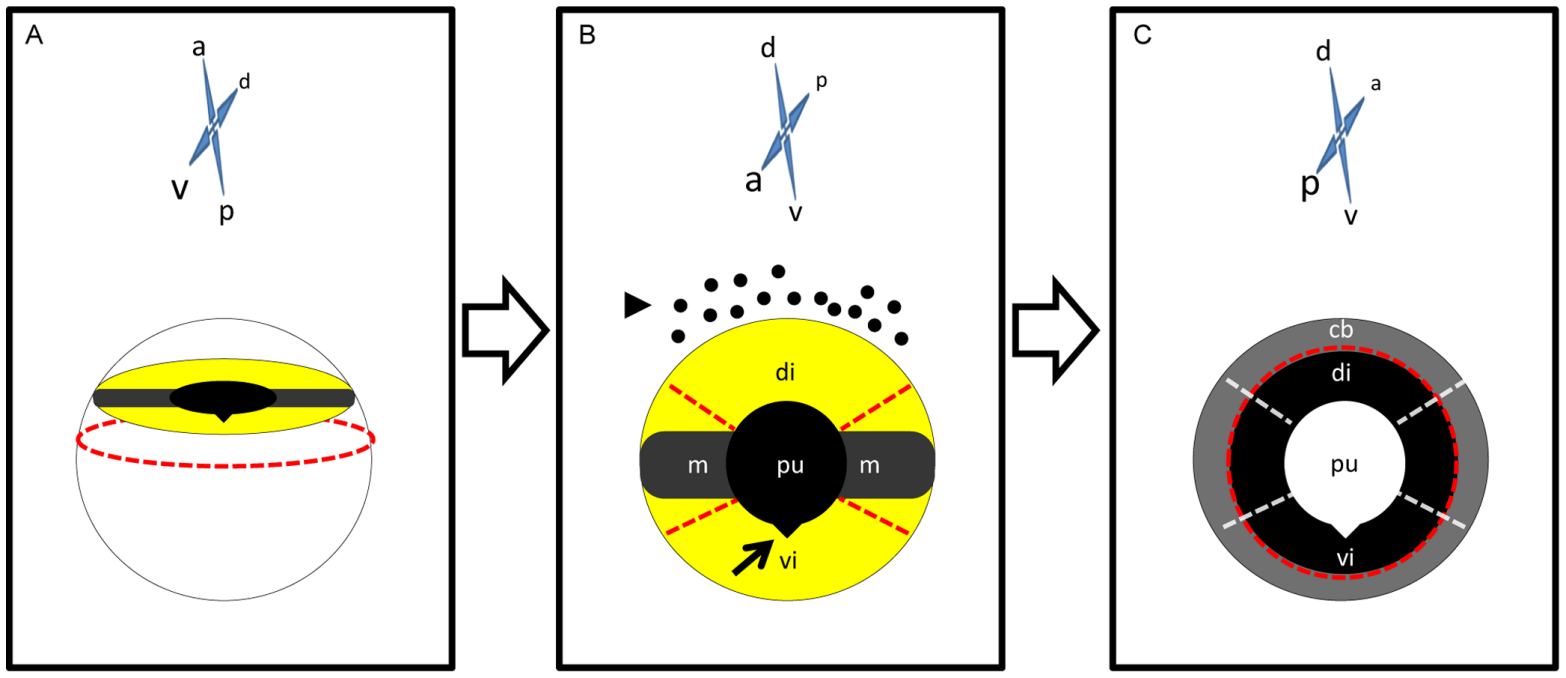
Diagram for collecting iris pieces. A. Whole eye ball with anterior side facing up and ventral facing the screen. Iris appears in the anterior side. Red dashed line indicates the plane that anterior and posterior sides are separated. B. Anterior view of a newt’s anterior part separated previously. Arrow head indicates black pigments present in the dorsal side of the eye. Arrow indicates the v-shaped pupil in the ventral side. These marks are indicative of the dorsoventral axis of the iris. Red dash lines indicate the separation of dorsal and ventral iris pieces performed while in the anterior view of the eye. C. Posterior view of a newt’s anterior part separated previously. Red dash lines indicate the separation of ciliary body and iris performed in this view. Transparent white dash lines indicate the separation of dorsal and ventral iris sectors performed in the anterior view. di: Dorsal iris sectors that have been isolated for the experiment, vi: Ventral iris sectors that have been isolated for the experiment. m: pigmented midline, cb: ciliary body, pu: pupil. Orientation in each panel is indicated above the illustrated eye parts, a: anterior side, d: dorsal side, v: ventral side, p: posterior side.

4 or 8 dpl newts were anesthetized in MS222. Whole eye balls were removed and placed in dishes filled with RNAlater® Solution (Applied Biosciences). Using fine scissors and tweezers, eye balls were dissected first by separating the anterior from the posterior part (Figure 1A) and then by removing remaining neural retina and the ciliary body from the anterior part (Figure 1C). Dorsal or ventral iris sectors were collected in approximately 135° of the whole iris leaving out a board area between dorsal and ventral side which has a black-colored pigmentation (Figure 1B). Dorsal and ventral sectors were then collected in microcentrifuge tubes filled with RNAlater® Solution. The tubes were briefly centrifuged and RNAlater® Solution was completely removed. RNA extraction was performed following TRIzol® Reagent protocol (Applied Biosciences) for 500 µl of reagent or the aqueous phase was transferred to RNA Clean & Concentrator™ (Zymo Research) columns and the recommended protocol was followed. Quality of isolated RNA was determined by Nanodrop 2000 spectrophotometer (Thermo Scientific). Good quality samples had A260/A280 ratio greater than 2 and a peak at 260 nm.

### Reverse Transcription Reaction (RT)

200 ng total RNA was used for RT reactions. First-strand cDNA synthesis kit (GE healthcare) was used following the recommended protocol for oligo(dt) primers. Half the volumes were used for negative RT reaction without using oligo(dt) primers and the samples were incubated for 5 min at 98°C for enzyme inactivation. All the samples had a clear band after Polymerase Chain Reaction (PCR) with RPL27 gene (housekeeping gene). Dorsal samples needed to be positive for TBX5 and negative for VAX2. Ventral samples needed to be positive for VAX2 and negative for TBX5.

### Quantitative Real-Time Polymerase Chain Reaction (qRT-PCR)

qRT-PCR was performed using iQ™ SYBR® Green Supermix (Bio-Rad) and Bio-Rad iCycler (Bio-Rad) following company’s protocol for 25 µl. Primer specificity was determined using melt curve analysis. An extra cycle of 6 sec was added to genes that were showing detectable signal from primer dimers and the temperature was determined by the melt curve (usually 2–4°C lower than the melting temperature; see methods below). Amplification cycles (Ct) of samples were compared to Ct of a standard curve created by the cDNA of the gene used. Gene expressions were then normalized to the expression of the reference gene (RPL27).

### Primers, PCR and qRT-PCR Settings

For the present study the following primers were used (written from 5′ to 3′): TBX5 Forward: CTGCCATGCCAGGGCGGTTG. TBX5 Reverse: GGTCGTGGGCAGGAGGTCCT. VAX2 Forward: TGTGCCAGCGCCACCTAACC. VAX2 Reverse: AGGTCCCCAAGCCGTACCCC. FGF10 Forward: GCTGTGCGTCACCAACTACT. FGF10 Reverse: TTGCTTTCTACGCCCCTCAC. NR2F5 Forward: CGGAACCTGAGCTACACCTG. NR2F5 Reverse: GGGAGATGAACCCCGTCAAG. UNC5B Forward: AGTCCAACCGGGGTGATCCTG. UNC5B Reverse: CATCTCGCTCTTGCCCATCTCC. NTN1 Forward: GGTTGCTCCACCCACTACAG. NTN1 Reverse: ACCATTCTCCAGCCTTGTCAG. RPL27 Forward: ATTTATGAAACCCGGGAAGG. RPL27 Reverse: CCAGGGCATGACTGTAAGGT.

PCR (performed with Premix Taq™ DNA polymerase (TaKaRa)) settings for TBX5∶40 cycles including 95°C for 30 sec, 65°C for 30 sec and 72°C for 30 sec. VAX2∶40 cycles including 95°C for 30 sec, 64°C for 30 sec, 72°C for 30 sec. RPL27∶40 cycles including 95°C for 30 sec, 55°C for 30 sec, 72°C for 30 secs. Last extension was 72°C for 10 mins for all the genes.

qRT-PCR settings for TBX5∶95°C for 3 mins, 40 cycles of 95°C for 30 sec, 65°C for 30 sec, 72°C for 30 sec and 85.5°C for 6 sec. VAX2∶95°C for 3 mins, 40 cycles of 95°C for 30 sec, 64°C for 30 sec, 72°C for 30 sec and 86.5°C for 6 sec. FGF10∶95°C for 3 mins, 40 cycles of 95°C for 30 sec, 57°C for 30 sec, 72°C for 30 sec and 84.5°C for 6 sec. NR2F5∶95°C for 3 mins, 40 cycles of 95°C for 30 sec, 57°C for 30 sec, 72°C for 30 sec. UNC5B: 95°C for 3 mins, 40 cycles of 95°C for 30 sec, 59°C for 30 sec, 72°C for 30 sec and 82°C for 6 sec. NTN1∶95°C for 3 mins, 40 cycles of 95°C for 30 sec, 57°C for 30 sec, 72°C for 30 sec and 82.5°C for 6 sec. RPL27∶95°C for 3 mins, 40 cycles of 95°C for 30 sec, 55°C for 30 sec, 72°C for 30 sec.

### Statistical Analysis for qRT-PCR Results

Statistical analysis was performed using two-way analysis of variance (ANOVA) and Student’s t-test for independed samples. Samples were run in triplicates (n = 3). Statistical significance was determined with 95% confidence (p<0.05). Equal variances for student’s t-test were assumed when Levene’s test p value was greater than 0.05.

### Newt Transcriptome, Data Mining and Functional Annotation

**Figure 2:**
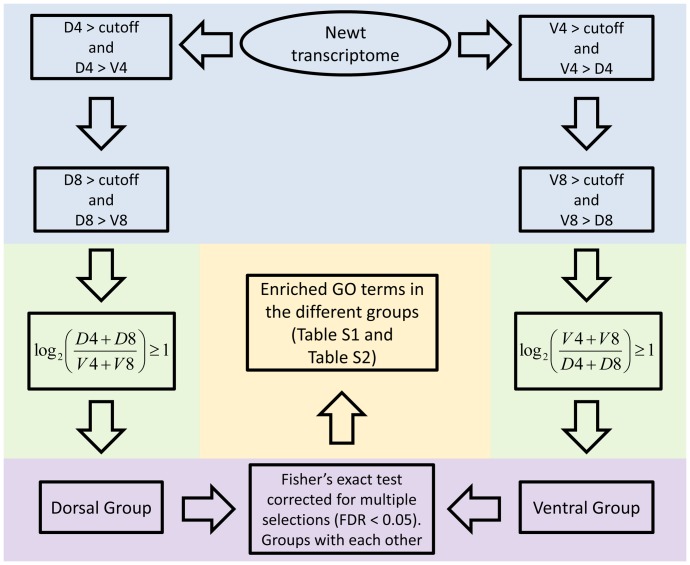
Workflow used to select transcripts for comparison of expression between the dorsal and ventral iris. Only transcripts expressed above the cutoff (see methods, light blue) and up-regulated at least 2-fold (light green) were included. Fisher’s exact test corrected with multiple selections (FDR <0.05) was used to compare the GO of the two groups (light purple). Enriched GO terms were found (light yellow).

**Figure 4:**
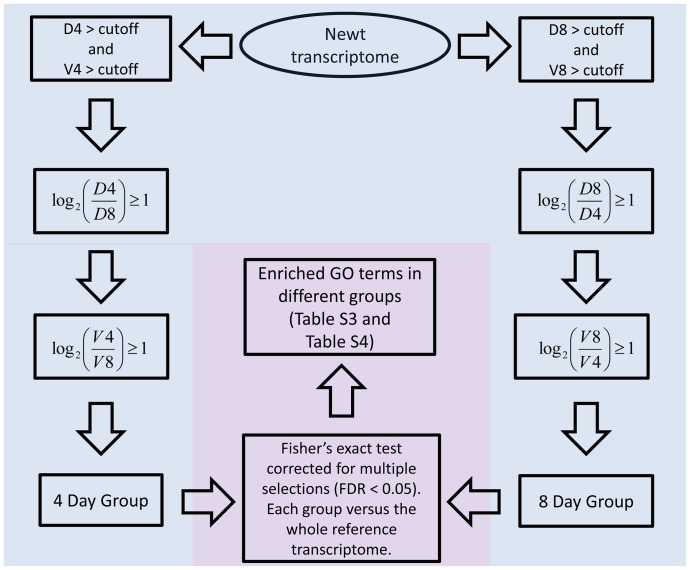
Workflow used to select transcripts for comparison of gene expression 4 and 8 days post-lentectomy. Only transcripts expressed above the cutoff (see methods) in the day of interest and up-regulated more than 2-fold in both dorsal and ventral iris (light blue) were considered. Fisher’s exact corrected for multiple selections (FDR <0.05) was used to compare the GO of the groups versus the remaining transcripts of the transcriptome. Enriched GO terms were found (light purple).

The newt transcriptome [8] was annotated with the BLAST2GO tool [9] using the nr database. We used a cutoff of (e−10) for sequence assignments, collected annotations and corresponding GO terms [10]. Transcripts were selected depending on their expression and location in the iris as described in Figure 2 for comparing dorsal iris versus ventral iris groups and as described in Figure 4 for comparing day 4 and day 8 groups. Fisher’s exact test corrected for multiple selections (feature available in BLAST2GO tool) was used for the different groups and statistically significant enriched GO terms were identified (FDR <0.05). Transcripts assigned to enriched terms were selected. Human homologues of those transcripts were found using the BLAST tool [11].

### RNA Expression Calculation

Illumina reads were mapped to the newt transcriptome using BWA [12]. Reads per kilobase per million mapped reads (RPKM) values were calculated for each transcript having at least one unique mapping read. Since intron/exon data are missing for the newt, we used a recently published microarray experiment for RPKM cutoff estimation [6]. We selected transcripts presented on the microarray having a valid spot structure (value for circularity >80%, intensity variation within the spot very small, flagged as valid) but lacking a significant signal during microarray analysis (signal to noise ratio (snr) <1, significant spots are used that have a snr ratio >3). We detected 101 spots to be valid for these parameters. Mapping of array coordinates to the transcriptome resulted in 81 non redundant individual transcripts. We assumed these candidates to be a good estimate for RPKM cutoff selection and calculated the average RPKM value for this transcript set for each investigated timepoint. We received a RPKM value of 0.64 for 4dv, 1.14 for 4dd, 1.13 for 8dv and 1.1 for 8dd. Illumina sequencing raw reads can be found in the NCBI sequence read archive under the accession: ERP001353 [8]. Assembled transcripts and annotation are located in the newtomics database [8], [13].

## Results and Discussion

### Transcriptome and Analysis Overview

The recently assembled newt transcriptome contains nearly 38,000 annotated genes [8]. After Illumina sequencing, the reads from the dorsal and ventral iris 4 and 8 dpl were mapped to the newt transcriptome and RPKM values were calculated. Since the newt genome is not yet available and reads that map to non-coding areas can not been found, we calculated the RPKM cutoff using micorarrays that were previously used during lens regeneration (see methods for more details). The cutoff in the RPKM values were 0.64 for ventral iris 4 dpl, 1.14 for dorsal iris 4 dpl, 1.13 for ventral iris 8 dpl and 1.1 for dorsal iris 8 dpl. Transcripts found to have RPKM more than the cutoff were considered to be expressed and were taken into account for the comparisons below.

### Dorsal Iris Shows Enrichment of Up-regulated Genes Involved in Cell Cycle, Cytoskeleton, Gene Expression and Immune Response

Since the major purpose of this study is to identify patterns of gene expression in the dorsal and ventral iris that might correlate with the ability of the dorsal iris for transdifferentiation, we first looked into transcripts that were regulated either in the dorsal or in the ventral iris at both collection points (4 dpl and 8 dpl). Analysis of the results was performed as outlined in Figure 2.

Interestingly, we only saw a handful of genes that are either exclusively expressed (no reads were mapped to them) in the dorsal or the ventral iris. This finding was not surprising because in the past our laboratory had seen, using limited expression data, that the ventral and dorsal irises show similar patterns of gene expression (see below).

Next, we investigated potential patterns of up-regulated genes. Specifically we asked which genes are consistently up-regulated in the dorsal or the ventral iris (both at 4 and 8 dpl). Fisher’s exact test with multiple testing correction for Gene Ontology (GO) from transcripts that are up-regulated at least 2 fold in dorsal iris 4 and 8 dpl compared to the ventral iris, and transcripts that are up-regulated at least 2 fold in ventral iris 4 and 8 dpl compared to the dorsal iris are shown in Table S1. Interestingly, we found that more GO terms are enriched in the dorsal samples than in the ventral. In particular, GO terms related to cell cycle, regulation of gene expression, cytoskeleton and immune response were overrepresented. In contrast, ventral samples, which generally generated fewer enriched GO terms, primarily showed GO terms related to transposons like RNA-directed DNA polymerase activity, RNA-directed DNA replication and DNA integration.

Although we did not find major qualitative differences in the expression patterns between dorsal and ventral irises, a clear-cut difference in the expression level of several genes were evident: GO terms related to cell cycle (indicating proliferation), cytoskeleton (indicating cell migration and morphological changes), transport of molecules and cytokinesis (indicating altered gene regulation), and immune response (indicating responsiveness to injury) were enriched at the site where lens regeneration commences. Strikingly, in these gene categories 609 genes were up-regulated in the dorsal iris and only 66 in the ventral iris, which will provide much needed insights into the mechanisms that allow the dorsal to “win” over the ventral iris.

### Cell Cycle-related Transcripts

Most of the factors related to cell cycle were up-regulated in the dorsal iris when compared to ventral iris (124/7) (Table 1). In detail:

**Table 1:** List of up-regulated (>2 times) transcripts related to cell cycle*.

Function	Dorsal						Ventral
Mitosis	CNTRL	PLK1	CLASP1	RAD21	NUSAP1	SMC4	NCOR1
	KIF11	Asun	STAG1	KATNB1	PARD3	TPX2	−
	TACC3	CDCA8	CEP120	KIF2C	ZWILCH	UBE2E1	−
	TUBB	CENPE	NCAPD2	NDC80	NEK3	ASPM	−
	RAB35	CENPF	NCAPG	SPC24	SGOL1	SMC3	−
	ROCK1	CEP192	ERCC6L	SPC25	DSCC1	AURKB	−
	NUP43	NEDD1	NCAPG2	KNTC1	SKA1	MAD2L1	−
	NUF2	NUMA1	−	−	−	−	−
tumor suppressor	PSMD10	XRN1	HBP1	MDM4	CENPF	MRPL41	PTEN
	CCAR1	APC	LIN9	E4F1	LIN9	TRRAP	−
Interphase	MNAT1	CUL4B	CDK2	MCM3	MCM7	LIN9	NR2F2
	CDC25A	CCNA2	POLA1	MCM4	UHRF1	HECTD3	−
	CDK1	CCNB1	POLE	MCM5	HEXIM1	MRPL41	−
	CENPF	CCNE2	MCM2	MCM6	DLGAP5	SMAD6	−
	RCC1	DSCC1	E4F1	APP	CHAF1A	NASP	−
	PLK4	SMARCA4	TAF2	RINT1	CHAF1B	−	−
DNA repair	HERC2	LIG1	GTF2H1	RAD1	TLK1	TOP2A	RNF8
	CLSPN	FANCI	H2AFX	CHEK1	−	−	RAD50
APC/C complex	ANAPC1	ANAPC7	FZR1+	CDC20	UBE2C	UBE2S	FZR1+
	ANAPC13	CDC27	−	−	−	−	−
proliferation	BOP1	CGRRF1	DST	HBP1	PHIP	SMARCA2	VASH1
	BMP2	NR2F2	FGF10	GTPBP4	PRDM4	STRADA	−
	BAX	DTYMK	CDCA7	−	−	−	−

GO:0007049 cell cycle; GO:0022403 cell cycle phase; GO:0000278 mitotic cell cycle; GO:0022402 cell cycle process; GO:0000087 M phase of mitotic cell cycle; GO:0051301 cell division; GO:0000279 M phase; GO:0007067 mitosis.

* Transcript names are from their human homologs.

+ Potential isoforms.

Mitosis: Table 1 shows factors related to all steps of mitosis including spindle formation, microtubule-associated, chromosomal movement and mitosis progression, which are up-regulated in dorsal samples. Interestingly, proteins that act as complexes are concomitantly up-regulated including NDC80 complex, cohesin complex and chromosomal passenger complex (CPC). NDC80 complex is essential for chromosome segregation and spindle formation [14], and NDC80, SPC24, SPC25 and NUF2 are up-regulated. Cohesin complex is required for the packing of the chromosomes [15], and STAG1, RAD21, SMC3, SMC4, PLK1 and SGOL1 are up-regulated. CPC is related to centromere functions during mitosis [16], and AURKB and CDCA8 are up-regulated.

Anaphase promoting complex/cyclosome (APC/C) is a complex that is instrumental for mitosis progression and division [17]. There are 8 APC/C-related transcripts which are up-regulated in dorsal samples: ANAPC1, ANAPC13, ANAPC7, CDC27, FZR1, UBE2S, CDC20 and UBE2C.

Interphase: Factors related to all phases of the interphase are up-regulated in dorsal samples. Cyclins and cyclin-dependent kinases that play a key role in G1/S, G2/M, G1 and S phases are up-regulated including CCNA2 [18], CCNB1 [19], CCNE2 [20], CDK1 [21] and CDK2 [22]. Proteins that act upon cyclins and cyclin-dependent kinases are up-regulated too, including MNAT1 [23], CDC25A [24] and HEXIM1 [25]. Factors that play a role in DNA synthesis during the S phase are up-regulated including POLA1, POLE [26], all the MCM complex (MCM2–7) [27], NASP [28], DSCC1 [29], CHAF1A and CHAF1B [30].

Tumor suppression, proliferation-related, p53/TP53-associated and RB1-associated proteins are up-regulated in dorsal iris. These proteins promote or repress proliferation and cell cycle. It has been previously shown that newt muscle cells re-enter the cell cycle after inactivation of RB. After entering the S phase these cells were resting in G2 phase. So, it was hypothesized that factors should be expressed, which promote the G2/M checkpoint after phosphorylation of RB [31]. Our data suggests that transcripts that regulate the G2/M transition and thereby proliferation include CDC25A, CDK1, CCNB1, the APC/C complex and PLK1.

Factors playing a role in DNA repair need to be seen in the context of cell proliferation and cell cycle, since the failure to repair DNA damage will prevent proliferation. In previous studies using qRT-PCR, it was shown that *rad1* is up-regulated at 3 and 5 dpl in the dorsal compared to ventral iris showing activation of robust DNA repair mechanism to prevent accumulation of mutations in dividing cells [6]. A similar pattern emerged in the current RNA-seq analysis: many factors that play a role in DNA repair are at least 2 fold up-regulated in the dorsal compared to the ventral iris. Among them RAD1 along with CHEK1, a kinase that has a key role in cell cycle arrest and apoptosis decisions after DNA damage [32].

### Gene Regulation-related Transcripts

It is expected that the process of transdifferentiation is marked by activation and regulation of many genes of the transcriptional apparatus. Indeed, we do find many genes related to gene regulation. Such genes are listed and categorized in Table 2 depending on the level of gene expression that they regulate (transcription, histone modification, RNA, translation). Again the majority of these genes show up-regulation in the dorsal iris (314 transcripts) and only a few show up-regulation in the ventral (39 transcripts).These include a number of proteins that play a role in the general transcriptional apparatus that transcribe all types of RNA including POLR1A, POLR2A, POLR1B, POLR1C, POLR1D, POLR2J, ZNRD1, GTF2H1, GTF2H2, MED12, MED23, MED24, ESF1, BRF1, TWISTNB and some factors from the CCR4-NOT complex (CNOT6, CNOT6L, CNOT4, CHAF1A, CHAF1B) [33]. Transcriptional factors and factors that link them to the basal transcriptional apparatus to regulate specific types or families of genes include ATF6 and CREB3L2, which activate certain genes upon stress [34], [35]. Furthermore, MYCBP and CDCA7 regulate MYC activity [36], [37], CBY1 inhibits Wnt via beta-catenin [38], NR2F2, RARA and RXRA are involved in gene activation after binding to retinoic acid [39], [40], [41], NR2C2 is a receptor that represses retinoic acid receptors [42], CYLD, RELB and NFKB2 are related to NF-kappaB pathway [43], [44], [45], RNF20 is involved in Hox gene activation [46], SMAD6 is a TGF-beta signaling-induced inhibitor of BMP signaling [47], [48], TBX5 is involved in dorsal eye patterning and in limb regeneration [49], [50], NR4A1 is a receptor found to play a role in liver regeneration [51], MDM4 inhibits p53 [52], and TEAD1 is involved in hippo pathway [53], are all up-regulated in the dorsal iris. It is noteworthy that VAX2 is up-regulated in the ventral iris, which is a major player in formation of ventral eye axis during embryogenesis [54]. It is interesting to speculate that the differential regulation of TBX5 and VAX2 in the dorsal and ventral iris demarcates their regenerative ability. TBX5 is expressed over 32-fold higher in the dorsal and VAX2 is expressed 32 fold higher in the ventral iris (see also below).

**Table 2:** List of up-regulated (>2 times) transcripts related to gene regulation*.

Function	Dorsal						Ventral
Transcription	ATF6	CNBP	POLR2A	FOXN2	ZNF3	SMAD6	NR2F1
	APP	CBY1	POLR2J	GATAD1	KLF15	MYCN	POLR1A+
	ARNTL	NR2F1	POLR1D	GTF2H1	KLF7	ZNFX1	HIPK2
	ARID4A	CBX1	DPF2	GTF2H2	LANCL2	NFKB2	VAX2
	KIAA2018	CHD7	E2F3	RAD54L2	LCOR	NR4A1	BRWD1
	BRMS1L	NR2F2	RNF20	HEXIM1	LIMD1	NR6A1	MYPOP
	BAHD1	CRTC2	UHRF1	HBP1	LMX1B	NR1H2	NR2F2
	MYCBP	CREB3L2	IKBKAP	HMX1	MEF2B	NR2C2	NFIB
	DENND4A	CYLD	ELP3	HIPK3	MED12	NFYB	NFE2L1
	MNAT1	POLR1A+	ELF2	HIPK2	MED23	ESF1	POLR2H
	CDCA7	ZNRD1	FOXJ2	IFT57	MED24	NFXL1	ZBED6
	POLR1C	PUF60	PHTF2	PHF6	PPARG	PWP1	PRDM2
	PRDM4	PQBP1	POLR1B	PHRF1	PHF12	PPARA	MLLT3
	XAB2	BUD31	RCOR2	SMARCA4	RELB	BRF1	ETS2
	PFDN5	LBH	RARA	TBX5	TFAP2B	TCF4	RAX
	CIAO1	MDM4	RXRA	BTAF1	TFAP2C	TFB1M	RGMB
	PHB2	RREB1	STAT6	TEAD1	E4F1	TAF12	LEO1
	ZEB1	ZGPAT	TWISTNB	TRIM33	TLE3	TAF2	SPEN
	ZFHX4	ZBTB5	WWTR1	TP53BP1	TLE4	TADA1	SOX6
	ZKSCAN1	ZIC1	RLF	SLC30A9	CNOT6	CHAF1B	TAF9B
	CNOT6L	CNOT4	CHAF1A	−	−	−	−
HistoneModifications	BRMS1L	RNF20	KAT5+	SETDB1	KDM2A	MLL3	ARID1A+
	ARID4A	TOPORS	MYSM1	SUV420H1	KDM2B	NAA16	KAT5+
	BRPF1	ENY2	RBBP7	KDM6B	KDM1B	NCOR1	KAT6A
	CHD4	EZH2	MLL	ARID1A+	MBTD1	NCOA3	MLL2
	DMAP1	KAT2B	WHSC1L1	MLL5	MTA3	PHF10	KDM5C
	WHSC1	SMARCA2	TRIM28	VPS72	PHF2	PHF12	NCOR1
	RBBP5	SMARCA4	TADA3	YEATS2	ASXL3	ZGPAT	−
	SIRT6	SMARCC2	TRRAP	YY1	−	−	−
RNA	XRN1	CPSF3	HEATR1	INTS6	WDR77	PPWD1	DHX29
	BOP1	DDX23	HNRNPAB	AQR+	MBNL2	PUF60	AQR+
	CARHSP1	DDX46	INTS1	CLASRP	MBNL1	PNPT1	PPIL3
	CSTF1	EBNA1BP2	INTS7+	CDK11B	PDCD11	PRPF8	INTS7+
	GEMIN4	EXOSC10	INTS9	DBR1	PPIH	XAB2	−
	SF1	SRSF11	SRRM1	RBM28	RP9	DDX41	−
	SFSWAP	SNRPA1	RSRC1	RBM5	NOP2	UTP11L	−
	SCAF1	UTP6	SRSF12	SARNP	RBFOX2	PUM1	−
	SNRPN	THOC2	THOC6	TFB1M	TFIP11	MPHOSPH10	−
	SMNDC1	THOC5	−	−	−	−	−
translation	MRPS18B	MRPL14	GFM1	EIF2B5	MRPL17	GTPBP4	EIF2B1+
	MRPS14	RPS27L	EEF2K	QRSL1	MRPL23	RARS2	RPS6KB2+
	MRPS5	CPEB2	EEF1E1	IARS	MRPL41	PDCD4	−
	MRPL15	DUS3L	EIF2D	MRPL12	MRPS9	TRUB2	−
	PUS10	PUS7	TRMT61A	TARS	GFM2	SRP14	−
	TRNAU1AP	RPL3	NSUN2	EIF2B1+	EEFSEC	EIF2B2	−
	DUS2L	RPS6KB2+	TRMT2A	−	−	−	−
miRNA	DICER1	MOV10	PNPT1	EIF2C3	TNRC6A	TNRC6C	−
Other	NEO1	AHSA1	AARSD1	A2M	DYNC2H1	SIRT5	PRKDC
	TACC3	PPIH	BAX	AKT2	EIF4ENIF1	PCSK1	HTT+
	CENPF	PPWD1	FGF10	TNFSF13B	MYO6	SERPINE1	INPPL1+
	C3	PFDN5	HTT+	BMP2	PEX1	NDFIP2	PTEN
	CCNA2	CDK2	MAPKAPK2	CCAR1	SORT1	NLK	RPS6KA4
	PSEN1	DISP1	RRAGC	RIPK1	MTOR	PHIP	PCM1
	EIF1AD	PPP3CB	RASSF8	ATP6AP2	TRIP11	INPPL1+	XPO5
	TBL3	TLR2	−	−	−	−	−

GO: 0010467 gene expression; GO:0010468 regulation of gene expression; GO:0006350 transcription; GO:0045449 regulation of transcription.

* Transcript names are from their human homologs.

+ Potential isoforms.

Factors that are involved in post-transcriptional regulation and in pre-mRNA maturation like splicing and alternative splicing and are up-regulated in the dorsal group include: GEMIN4 [55], DDX23, DDX41, AQR, PPWD1, XAB2, SRRM1, TFIP11 [56], DDX46 [57], CLASRP, CDK11B [58], DBR1 [59], WDR77 [60], MBNL2, MBNL1 [61], PPIH [62], PUF60 [63], PRPF8 [64], RBFOX2 [65], RBM28 [66], RBM5 [67], RSRC1 [68], SRSF12 [69], SRSF11 [70], SFSWAP [71], SCAF1 [72] and SMNDC1 [73], or other ways of pre-mRNA maturation using CSTF1 [74], CPSF3 [75] and EXOSC10 [76]. Factors that play a role in ribosomal RNA maturation include BOP1 [77], EBNA1BP2 [78], HEATR1 [79], PDCD11 [80], UTP11L, UTP6 [81] and MPHOSPH10 [82]. Factors that play role in the stability and transport of the RNA include CARHSP1 [83], PUM1 [84] and part of the TREX complex (THOC2, THOC5, and THOC6) [85].

Protein complexes and related factors involved in histone modifications and are up-regulated in dorsal group include the SIN3A/HDAC1 complex (BRMS1L, ARID4A, TOPORS, RBBP7, NCOR1 and SMARCC2), the NuRD complex (CHD4, RBBP7, MTA3, TRIM28 and ZGPAT) [86],the NuA4 complex (DMAP1, KAT5, TRRAP and VPS72) [87], the PRC2/EED-EZH2 complex (EZH2 and RBBP7) [88], the MLL1/MLL complex (MLL and RBBP5) [89], and the SWI/SNF complex (BAF complexes) (ARID1A, PHF10, SMARCA2, SMARCA4 and SMARCC2) [90], and are all up-regulated in the dorsal iris.

Likewise, most of the transcripts that were identified to act on translation (and miRNA processing and function) are up-regulated in the dorsal iris. Only 1 transcript, eIF2B, was found to be up-regulated in the ventral iris.

### Cytoskeleton-related Transcripts

Table 3 shows transcripts that are up-regulated in dorsal iris at least 2 fold than the ventral iris and the opposite. Most of the transcripts are shown to be up-regulated in the dorsal iris (134/18).

**Table 3:** List of up-regulated (>2 times) transcripts related to cytoskeleton*.

Function	Dorsal						Ventral
microtubule	CNTRL	TUBA1A	CEP120	DNAH7	KIF22	KNTC1	CLIC5
	SSNA1	BBS2	CYLD	TUBGCP6	KIF23	DYNLT1	MARK1
	CHEK1	TUBB	DYNC1I2	HTT+	KIF2C	AURKB	HTT+
	CENPF	TUBB4B	DYNC2H1	IFT57	KIF14	LYST	PCM1
	KIF11	CAMSAP3	DYNC1LI1	KATNB1	KIF20A	MAP1B	KIF1B
	PLK1	CEP170	CLIP2	KIAA1279	KIF13A	MACF1	KIAA0284
	APC	CLASP1	DNM2	KIF13B	NDC80	MAST2	NCOR1
	PSKH1	SMC3	CCNB1	CDC27	TUBB3	PLK4	−
	ARL2BP	RIF1	MAD2L1	CEP350	TUBD1	SNTB2	−
	TPX2	ASPM	NUP85	CEP192	ZNF415	SKA1	−
	CDCA8	CENPE	RANGAP1	CBX1	NEDD1	DCX	−
	NIN	NINL	NUMA1	NUSAP1	SHROOM2	SHROOM3	−
	RAB3IP	AURKA	−	−	−	−	−
actin	WASH1	CTNNA1	MYO1E	MYO5A	PLEK2	VASP	ACTA2
	ARPC1B	INTS6	MYO9A	MYO7A	DIAPH1	WDR1	INPPL1+
	ARPC5L	IQGAP1	MTSS1	PPP1R9A	SSH2+	INPPL1+	SSH2+
	ACTR6	KLHL3	MYO1G	MYO1D	RAB3IP	SNTB2	RDX
	SCIN	MYO10	MYO6	PDLIM5	IQGAP2	SYNE1	TPM1
	CNN2	LANCL2	MYO9B	MACF1	ROCK1	−	UTRN+
Other	MPP1	GAN	LMNB1	SYNM	NES	BAG1	DES
	FERMT3	DLGAP5	NF2	UTRN+	TNS1	UBR4	RAI14
	GNE	RAPH1	NF1	CORO1A	ANK3	SLC26A5	SLC4A1
	APP	FRMD6	TRIB2	PTPN14	SLC30A9	STOML2	MICALL2
	PSEN1	−	−	−	−	−	−

GO:0005856 cytoskeleton; GO:0015630 microtubule cytoskeleton; GO:0044430 cytoskeletal part.

* Transcript names are from their human homologs.

+ Potential isoforms.

Microtubules-associated: In this category some of the transcripts are related to the cell cycle like in spindle formation and chromosome movement and we have discussed them previously. Other transcripts that are up-regulated in the dorsal group have a role during signal transduction: APC negatively regulates Wnt signaling [91], MACF1 positively regulates Wnt signaling [92], CYLD and MAST2 positively regulates NF-kappaB pathway [93]. Transcripts involved in microtubule organization and stability include CAMSAP3 [94], CEP170 [95], CLASP1 [96], KATNB1 [97], KIAA1279 [98], MAP1B [99], NIN [100], NINL [101] and NUSAP1 [102]. Transcripts involved in transport of molecules include DYNC1I2 [103], DYNC1LI1 [104], DYNC2H1 [105], DNM2 [106], HTT [107], LYST [108], DYNLT1 [109] and PSKH1 [110], in cilia formation include BBS2 [111], DNAH7 [112] and IFT57 [113], in cell shape and movement include ELMO2 [114] and GAN [115].

Actin-related: Transcripts up-regulated in the dorsal group and included in this category play roles in actin polymerization and organization in order to support cell shape, movement and cell adhesion with the extracellular matrix, and the linkage of actin with other proteins in order to facilitate transport or contraction. WASH1 [116], ARPC1B, ARPC5L [117], SSH2 [118] SCIN [119], IQGAP1 [120], PPP1R9A [121] and DIAPH1 [122] are playing roles in actin polymerization and organization. CNN2, MYO10, MYO1E, MYO9A, MYO1G, MYO6, MYO9B, MYO7A, MYO1D and ROCK1 are involved in contraction [123], [124]. CTNNA1 and MTSS1 are related to cell adhesion with the extracellular matrix and cell-cell contact [125], [126]. KLHL3 and MYO5A are playing roles in molecular transport [127], [128]. PLEK2 and VASP are related to cell movement [129], [130] and SYNE1 and WDR1 are linking cytoskeleton with other proteins [131], [132].

In addition to factors that are actin or tubulin-related, Table 3 shows other proteins that are related to cell adhesion, movement and linkage of plasma membrane with cytoskeleton that are up-regulated in the dorsal iris. For lens regeneration, cell adhesion and locomotion is very important since PECs need to change their environmental behavior to transdifferentiate and change their cell fate. Previous studies have found that extracellular matrix is being remodeled and matrix metalloproteinases are up-regulated already 1 day post-lentectomy to prepare the environment for the onset of lens regeneration [6]. Our data clearly show changes in the molecules that determine the interaction of PECs with the environment and remodeling of cytoskeleton components and networks of PECs. Another interesting aspect is that many factors involved in tumor metastasis are up-regulated in the dorsal iris which indicates a role of these molecules at the onset of cell locomotion.

### Immunity-related Transcripts

Most of the transcripts related to immune response are up-regulated in the dorsal iris samples (Table 4). Only 2 transcripts were up-regulated in the ventral iris versus 37 that were up-regulated in the dorsal iris. Factors in this category regulate NF-kappaB activity among others and include SIVA1 [133], CHUK, NFKB2, TLR7 [134] and RELB. Factors involved in immune cells activation and migration include TNFSF13B [135], CD97 [136], GPR183 [137], ENPP2 [138], DCLRE1C [139] and TLR2 [140]. Complement component -related transcripts include C1S, C3, C1QB and C1QBP and other factors involved in cytokine secretion and inflammation include CCL5, DDX58, STAT6 and ZEB1.

**Table 4:** List of up-regulated (>2 times) transcripts related to immune response*.

Dorsal								Ventral
A2M	TNFSF13B	TUBB4B	CD97	CCL5	C3	C1QBP	DDR1	TIMM50
SIVA1	TUBB	CTNNBL1	CADM1	C1S	C1QB	CHUK	TOPORS	INPPL1+
RELB	ENPP2	STAT6	PPP3CB	INPPL1+	NCF2	GAB2	SMAD6	−
SYK	FCN1	TLR2	RARA	PSEN1	NFKB2	LYST	DDX58	−
ZEB1	GPR183	TLR7	INPP5D	DCLRE1C	−	−	−	−

GO:0006955 immune response.

* Transcript names are from their human homologs.

+ Potential isoforms.

The role of immune response and its involvement in the initiation of regeneration has been extensively investigated in the past [141], [142]. It has been hypothesized that molecules involved in the regulation of the immune response have a novel role in regeneration or that the immune response itself is crucial for regeneration. The issue has not been settled yet. Nevertheless, complement components seem to be important for liver regeneration [143] and have also found to be expressed in limb and lens regeneration [144], [145]. The present results provide strong evidence of a crucial role of injury response in regeneration, which needs to be investigated further.

### Transposon-related Transcripts

Interestingly, transposons are the only transcripts that are enriched in ventral compared to dorsal samples (Table S2). Transposons have many types and they do not have an assigned biological function. They can be transcribed, reverse-transcribed and integrated back to the genome (retrotransposons) or not. So far, we have no specific role of transposons in regeneration (or rather, inhibition of it?). Since transposon-related transcripts are enriched in ventral samples it would be interesting to learn more about a specific role in repressing specific programs.

### Highly Regulated Transcripts

Summing up, we have identified patterns of gene expression that are predominant in the dorsal iris. Genes that are involved in cell cycle, gene regulation, cytoskeleton and immune response show a graded expression along the dorsal/ventral iris. Thus, our study is the first to show how the dorsal iris differs from the ventral iris, and how specific patterns of gene expression correlates with the dorsal iris’ regenerative ability. Comparisons comprised two critical time points, 4 and 8 dpl. Interestingly, comparison of gene expression patterns at each time point separately, recapitulates our primary finding that most transcripts of these gene categories are up-regulated at 4 dpl in the dorsal iris in comparison to 4 dpl in the ventral iris, or in 8 dpl in the dorsal iris in relation to 8 dpl in the ventral iris. In Tables 5 and 6 we show a selected group of genes to exemplify this point. The tables also allow a view on the top regulated transcripts. It becomes clear that a few of them are either dorsal-specific or ventral-specific. Only 3 transcripts were found to be exclusively present in the dorsal iris. These transcripts correspond to protein-1 like (ras associated and pleckstrin domains-containing), transmembrane protein 185A-like (TMEM family) and to chromatin assembly factor 1 (CAF1). Other transcripts that show very high expression in dorsal iris were TBX5, TMEM185A, E3 ubiquitin-protein ligase HERC2-like (HERC2) (>32 times), TMEM116, ephrin–B2, and netrin receptor (UNC5B) (>16 times). In the ventral iris, except transposons, we find that netrin-1 (NTN1), nuclear receptor 2F5-like (NR2F5), and VAX2 are expressed 32 times higher than in the dorsal iris. The function of TBX5 and VAX2 were discussed above, but it is interesting to note here that they might provide a dorsal or ventral identity to the adult iris. Currently, it is not known to what extent these genes control regeneration but functional assays will settle this issue. Nevertheless, TBX5 and VAX2 can be used as markers for dorsal and ventral iris, respectively. Interestingly, we find NTN1 in the ventral iris but its receptor (UNC5B) in the dorsal iris. Likewise, we find ephrin-B2 in the dorsal and its receptor in the ventral iris, which might reveal a so-far unsuspected communication between dorsal and ventral iris. Despite its role in axon guidance, NTN1 has also been shown to inhibit leukocyte migration. Thus, NTN1 up-regulation might protect injured tissues [146]. UNC5B is responsible for apoptosis and because NTN1 is up-regulated by p53 it is considered as an oncogene [147]. Ephrin receptors activated by ephrins have been shown to inhibit signaling by oncogenes. The case of TMEM proteins is interesting as well. Even though not much is known for TMEM185A and TMEM116, TMEM16F is known to form a Ca^2+^-activated channel, which plays a role in blood coagulation that is also mediated by thrombin activation [148]. In turn, blood coagulation has been implicated in the induction of lens regeneration from the dorsal iris [142].

**Table 5:** A selected list of 50 transcripts highly up-regulated in the dorsal iris.

transcript ID	Annotation (Blastx against nr)	v4	d4	v8	d8	log2Fc4	log2Fc8	log2Fc
transcript114225	LOW QUALITY PROTEIN: ras-associated and pleckstrin homology domains-containing protein 1-like [Xenopus (Silurana) tropicalis]#	0.000	2.987	0.000	1.296	#DIV/0!	#DIV/0!	######
transcript89311	transmembrane protein 185A-like [Anolis carolinensis]#	0.000	6.490	0.000	1.292	#DIV/0!	#DIV/0!	######
transcript53947	similar to Chromatin assembly factor 1 subunit B [Canis familiaris]#	0.000	5.182	0.000	1.986	#DIV/0!	#DIV/0!	######
transcript75898	pG1 protein [Lactobacillus jensenii 269-3] #	0.331	5.828	0.151	19.812	4.140	7.038	5.735
transcript41516	LOW QUALITY PROTEIN: probable E3 ubiquitin-protein ligase HERC2-like [Xenopus (Silurana) tropicalis]#	0.000	4.070	0.193	2.527	#DIV/0!	3.713	5.098
transcript28206	regeneration blastema forelimb-specific Tbx [Notophthalmus viridescens]#	0.272	4.549	0.248	12.007	4.062	5.595	4.990
transcript14449	similar to TMEM116 protein [Gallus gallus]#	0.335	1.492	0.485	20.092	2.156	5.373	4.719
transcript12545	hypothetical protein LOC432274 [Xenopus laevis]	0.000	3.858	0.253	1.988	#DIV/0!	2.976	4.532
transcript63962	hypothetical protein LOC495396 [Xenopus laevis]	0.290	4.756	0.000	1.765	4.038	#DIV/0!	4.493
transcript80306	ephrin-B2 [Taeniopygia guttata]#	0.302	4.602	0.358	8.955	3.930	4.645	4.361
transcript87396	programmed cell death 2 [Xenopus laevis]	0.140	2.293	0.127	3.168	4.038	4.637	4.354
transcript89253	transmembrane protein 116-like [Meleagris gallopavo]#	0.163	1.691	0.653	14.078	3.374	4.430	4.272
transcript59308	DEP domain-containing protein 1B-like isoform 1 [Nomascus leucogenys]#	0.106	3.145	0.194	2.270	4.886	3.548	4.172
transcript15984	nuclear pore membrane glycoprotein 210 precursor [Mus musculus]#	0.000	1.977	0.183	1.155	#DIV/0!	2.658	4.097
transcript88594	similar to Thimet oligopeptidase [Canis familiaris]#	0.000	3.005	0.298	1.991	#DIV/0!	2.740	4.068
transcript82136	WD repeat and FYVE domain-containing protein 3 isoform 1 [Monodelphis domestica]#	0.000	2.541	0.252	1.684	#DIV/0!	2.740	4.068
transcript92162	netrin receptor UNC5B-like [Xenopus (Silurana) tropicalis]#	0.000	2.333	0.476	5.587	#DIV/0!	3.552	4.056
transcript89179	apoptosis-inducing factor 2-like [Anolis carolinensis]#	0.000	4.743	0.387	1.246	#DIV/0!	1.686	3.951
transcript22057	dihydroxyacetone kinase 2 [Taeniopygia guttata]#	0.000	2.147	0.282	2.094	#DIV/0!	2.892	3.911
transcript88286	lysophospholipid acyltransferase 5-like [Xenopus (Silurana) tropicalis]#	0.132	2.776	0.181	1.922	4.394	3.412	3.909
transcript103122	LOW QUALITY PROTEIN: laminin subunit alpha-4-like [Xenopus (Silurana) tropicalis]#	0.000	5.539	0.513	2.030	#DIV/0!	1.985	3.884
transcript57122	calmodulin-regulated spectrin-associated protein 3 [Danio rerio]#	0.000	4.118	0.497	2.953	#DIV/0!	2.570	3.830
transcript65968	biphenyl hydrolase-like (serine hydrolase) [Xenopus laevis]#	0.000	3.044	0.470	3.573	#DIV/0!	2.928	3.817
transcript87765	ras and Rab interactor 2-like [Meleagris gallopavo]#	0.278	4.391	0.127	1.320	3.979	3.378	3.816
transcript115898	serine/threonine-protein kinase PLK4 [Xenopus (Silurana) tropicalis]#	0.000	3.900	0.467	2.426	#DIV/0!	2.378	3.761
transcript105338	epithelial cell transforming sequence 2 oncogene [Xenopus (Silurana) tropicalis]#	0.000	2.945	0.303	1.124	#DIV/0!	1.892	3.748
transcript55938	hypothetical protein [Gallus gallus]#	0.000	7.267	0.811	3.277	#DIV/0!	2.015	3.701
transcript23291	LOW QUALITY PROTEIN: serine/threonine-protein kinase WNK1-like [Anolis carolinensis]#	0.210	3.866	0.240	1.850	4.201	2.949	3.668
transcript93294	39S ribosomal protein L15, mitochondrial precursor [Xenopus laevis]#	0.155	4.783	0.353	1.574	4.948	2.155	3.645
transcript11364	glycerol kinase [Candidatus Liberibacter americanus]#	4.046	7.288	0.580	50.265	0.849	6.436	3.637
transcript111723	adenylate cyclase type 6 isoform 2 [Equus caballus]#	0.285	7.856	0.519	1.350	4.786	1.378	3.517
transcript63503	coiled-coil domain-containing protein 85C-like [Anolis carolinensis]#	0.000	3.369	0.567	3.051	#DIV/0!	2.428	3.501
transcript56063	hypothetical protein RAYM_09754 [Riemerella anatipestifer RA-YM]#	1.421	22.491	0.678	1.115	3.984	0.719	3.492
transcript63410	signal peptide, CUB and EGF-like domain-containing protein 3 [Monodelphis domestica]#	0.000	2.531	0.479	2.609	#DIV/0!	2.445	3.423
transcript59917	cell cycle regulator Mat89Bb homolog [Sus scrofa]#	0.000	8.811	1.121	3.103	#DIV/0!	1.469	3.409
transcript95772	hypothetical protein [Gallus gallus]#	0.113	2.902	0.413	2.646	4.679	2.679	3.398
transcript23822	UPF0679 protein C14orf101-like [Sus scrofa]#	0.000	6.341	0.880	2.745	#DIV/0!	1.641	3.368
transcript109593	mesoderm development candidate 1 [Taeniopygia guttata]#	0.261	3.091	0.298	2.522	3.564	3.081	3.327
transcript84524	unnamed protein product [Tetraodon nigroviridis]#	0.000	1.879	0.356	1.497	#DIV/0!	2.073	3.246
transcript25525	fas apoptotic inhibitory molecule 1-like [Anolis carolinensis]#	0.000	7.291	0.973	1.763	#DIV/0!	0.857	3.218
transcript47004	LOW QUALITY PROTEIN: matrix-remodeling-associated protein 5-like [Pongo abelii]#	0.000	4.089	0.690	2.278	#DIV/0!	1.722	3.205
transcript83773	hypothetical protein [Ornithorhynchus anatinus]#	0.205	6.858	0.933	3.327	5.066	1.833	3.162
transcript80809	HIV-1 tat interactive protein [Danio rerio]#	0.221	3.551	0.402	2.017	4.009	2.325	3.160
transcript101040	LOW QUALITY PROTEIN: protein NEDD1-like [Anolis carolinensis]#	0.000	2.837	0.525	1.755	#DIV/0!	1.740	3.128
transcript83315	ras GTPase-activating-like protein IQGAP2 [Xenopus (Silurana) tropicalis]#	0.000	3.094	0.537	1.424	#DIV/0!	1.407	3.073
transcript48784	30S ribosomal protein S14 [Chryseobacterium gleum ATCC 35910]#	0.761	10.280	0.644	1.509	3.756	1.228	3.069
transcript19175	hyaluronan synthase 2-like [Anolis carolinensis]#	0.205	7.412	1.975	10.789	5.175	2.450	3.062
transcript90710	vaccinia related kinase 1 [Xenopus laevis]#	0.311	2.960	0.213	1.315	3.252	2.629	3.030
transcript119283	neurobeachin-like protein 1-like [Anolis carolinensis]#	0.000	5.855	0.889	1.320	#DIV/0!	0.570	3.013
transcript104553	exocyst complex component 1-like isoform 1 [Anolis carolinensis]#	0.29	4.137	0.47	1.884	3.8202	2.011	2.985

v4: RPKM value of ventral iris 4 dpl, d4: RPKM value of dorsal iris 4 dpl, v8: RPKM value of ventral 8 dpl, d8: RPKM value of dorsal iris 8 dpl, log_2_Fc4: fold expression at 4 dpl between dorsal and ventral iris, log_2_Fc8: fold expression at 8 dpl between dorsal and ventral iris, log_2_Fc: fold expression between dorsal and ventral iris at both the days.

**Table 6:** A selected list of 50 transcripts highly up-regulated in the ventral iris.

transcript ID	Annotation (Blastx against nr)	v4	d4	v8	d8	log2Fc4	Log2Fc8	Log2Fc
transcript31815	netrin-1-like [Xenopus (Silurana) tropicalis]#	5.052	0.141	7.897	0.066	−5.166	−6.892	−5.965
transcript93602	retrotransposable element Tf2 155 kDa protein type 1-like, partial [Xenopus (Silurana) tropicalis]#	2.200	0.000	3.177	0.124	#NUM!	−4.678	−5.437
transcript32521	ventral anterior homeobox 2a-like [Xenopus (Silurana) tropicalis]#	6.383	0.271	9.860	0.122	−4.560	−6.336	−5.371
transcript26555	nuclear receptor subfamily 2 group F member 5-like [Xenopus (Silurana) tropicalis]#	6.299	0.378	14.165	0.158	−4.057	−6.482	−5.253
transcript73289	hypothetical protein LOC100486110 [Xenopus (Silurana) tropicalis]#	8.643	0.196	2.039	0.404	−5.464	−2.336	−4.155
transcript99097	hypothetical protein LOC100485130 [Xenopus (Silurana) tropicalis]#	19.132	0.951	3.079	0.708	−4.331	−2.121	−3.744
transcript45528	ORF2-encoded protein [Danio rerio]#	9.423	0.436	1.574	0.449	−4.434	−1.808	−3.635
transcript14061	similar to reverse transcriptase-like protein [Strongylocentrotus purpuratus]#	10.385	0.315	2.696	0.811	−5.043	−1.732	−3.538
transcript66096	retrotransposable element Tf2 155 kDa protein type 1-like [Danio rerio]#	6.047	0.567	3.939	0.292	−3.413	−3.752	−3.538
transcript97196	ATP-binding cassette, sub-family G (WHITE), member 2 [Xenopus laevis]#	4.516	0.424	2.059	0.218	−3.413	−3.237	−3.356
transcript19896	CR1-3 [Lycodichthys dearborni]#	8.640	0.481	3.807	0.744	−4.167	−2.356	−3.345
transcript106073	sorting nexin-25 [Monodelphis domestica]#	2.248	0.246	3.760	0.381	−3.191	−3.304	−3.261
transcript67285	zinc finger protein 850-like [Monodelphis domestica]#	1.431	0.209	1.305	0.081	−2.776	−4.015	−3.240
transcript8700	similar to ORF2-encoded protein [Strongylocentrotus purpuratus]#	6.676	0.000	3.119	1.103	#NUM!	−1.500	−3.151
transcript116527	retrotransposable element Tf2 155 kDa protein type 1-like [Anolis carolinensis]#	4.011	0.465	3.874	0.479	−3.109	−3.015	−3.062
transcript8488	hypothetical protein LOC100494670 [Xenopus (Silurana) tropicalis]#	8.977	0.985	2.830	0.515	−3.188	−2.459	−2.977
transcript44589	reverse transcriptase [Anguilla japonica]#	14.281	2.024	6.449	0.616	−2.819	−3.387	−2.973
transcript41350	hypothetical protein LOC100496475 [Xenopus (Silurana) tropicalis]#	15.178	1.601	2.794	0.731	−3.245	−1.935	−2.946
transcript56731	hypothetical protein LOC100494422 [Xenopus (Silurana) tropicalis]#	12.385	1.216	3.181	0.819	−3.349	−1.957	−2.935
transcript15059	reverse transcriptase [Danio rerio]#	16.797	1.881	3.116	0.724	−3.159	−2.106	−2.934
transcript30275	cleavage stimulation factor subunit 2-like [Homo sapiens]#	7.765	0.900	2.351	0.442	−3.109	−2.411	−2.914
transcript3811	pol protein [Salamandra salamandra]#	15.064	1.665	5.586	1.192	−3.178	−2.229	−2.854
transcript19227	similar to reverse transcriptase-like protein, partial [Strongylocentrotus purpuratus]#	8.679	0.292	2.690	1.281	−4.891	−1.070	−2.853
transcript41219	hypothetical protein LOC100493707 [Xenopus (Silurana) tropicalis]#	7.539	0.842	1.994	0.485	−3.162	−2.040	−2.845
transcript21599	SPARC-related modular calcium-binding protein 1-like [Anolis carolinensis]#	5.688	1.029	13.814	1.709	−2.467	−3.015	−2.832
transcript67066	phosphatidylinositol-3,4,5-trisphosphate 5-phosphatase 2-like [Anolis carolinensis]#	8.278	0.680	3.421	0.964	−3.606	−1.828	−2.832
transcript40028	reverse transcriptase-like protein [Salmo salar]#	8.686	0.839	4.019	0.951	−3.372	−2.079	−2.827
transcript84492	similar to Family with sequence similarity 116, member A [Ornithorhynchus anatinus]#	6.781	0.431	1.496	0.741	−3.975	−1.015	−2.820
transcript18114	Pro-Pol-dUTPase polyprotein [Mus musculus]#	3.825	0.234	3.326	0.783	−4.032	−2.087	−2.814
transcript29937	reverse transcriptase [Chironomus tentans]#	10.035	0.840	2.872	0.999	−3.578	−1.523	−2.811
transcript5182	similar to LReO_3 [Strongylocentrotus purpuratus]#	20.890	2.839	6.570	1.098	−2.879	−2.582	−2.802
transcript71146	putative nuclease HARBI1 [Xenopus (Silurana) tropicalis]#	47.020	5.281	10.514	2.979	−3.154	−1.820	−2.800
transcript1139	sushi, von Willebrand factor type A, EGF and pentraxin domain-containing protein 1-like [Xenopus (Silurana) tropicalis]#	27.032	2.851	7.238	2.099	−3.245	−1.786	−2.791
transcript57867	endonuclease/reverse transcriptase [Sus scrofa]#	5.817	0.249	1.211	0.771	−4.545	−0.652	−2.785
transcript78186	reverse transcriptase-like protein-like [Saccoglossus kowalevskii]#	15.112	1.917	2.519	0.670	−2.979	−1.910	−2.768
transcript2222	reverse transcriptase-like protein [Paralichthys olivaceus]#	14.076	1.403	3.805	1.237	−3.327	−1.622	−2.760
transcript5593	Myb protein P42POP, isoform CRA_a [Mus musculus]#	13.400	1.539	3.506	0.967	−3.122	−1.858	−2.754
transcript899	hypothetical protein TcasGA2_TC015886 [Tribolium castaneum]#	10.490	0.880	2.036	0.982	−3.576	−1.051	−2.750
transcript51721	hypothetical protein LOC100488716 [Xenopus (Silurana) tropicalis]#	4.344	0.000	1.585	0.882	#NUM!	−0.845	−2.748
transcript7725	hypothetical protein LOC734400 [Xenopus laevis]#	9.043	0.907	2.479	0.815	−3.317	−1.605	−2.742
transcript108620	hypothetical protein LOC100497892 [Xenopus (Silurana) tropicalis]#	17.822	2.260	3.565	0.953	−2.980	−1.904	−2.735
transcript70652	hypothetical protein LOC100492542 [Xenopus (Silurana) tropicalis]#	7.011	0.825	2.004	0.532	−3.087	−1.915	−2.733
transcript15129	similar to transposase [Strongylocentrotus purpuratus]#	7.680	0.917	4.192	0.886	−3.066	−2.242	−2.718
transcript1001	hypothetical protein LOC100490320 [Xenopus (Silurana) tropicalis]#	44.292	5.312	9.908	2.944	−3.060	−1.751	−2.715
transcript7879	NADH dehydrogenase (ubiquinone) 1 alpha subcomplex, 4 b [Xenopus laevis]#	1521.4	153.4	130.6	98.732	−3.310	−0.403	−2.712
transcript112029	similar to CR1 Danio rerio 2 reverse transcriptase isoform 3 [Strongylocentrotus purpuratus]#	17.674	1.804	3.208	1.394	−3.293	−1.202	−2.707
transcript68620	hypothetical protein LOC100497926 [Xenopus (Silurana) tropicalis]#	5.412	0.790	3.565	0.611	−2.776	−2.545	−2.680
transcript1148	hypothetical protein LOC100493982 [Xenopus (Silurana) tropicalis]#	17.683	2.600	5.544	1.056	−2.766	−2.392	−2.667
transcript338	reverse transcriptase-like protein [Takifugu rubripes]#	364.357	50.283	86.144	20.794	−2.857	−2.051	−2.664
transcript1216	hypothetical protein LOC100495475, partial [Xenopus (Silurana) tropicalis]#	8.246	0.355	2.898	1.419	−4.537	−1.030	−2.651

v4: RPKM value of ventral iris 4 dpl, d4: RPKM value of dorsal iris 4 dpl, v8: RPKM value of ventral 8 dpl, d8: RPKM value of dorsal iris 8 dpl, log_2_Fc4: fold expression at 4 dpl between dorsal and ventral iris, log_2_Fc8: fold expression at 8 dpl between dorsal and ventral iris, log_2_Fc: fold expression between dorsal and ventral iris at both the days.

**Figure 3:**
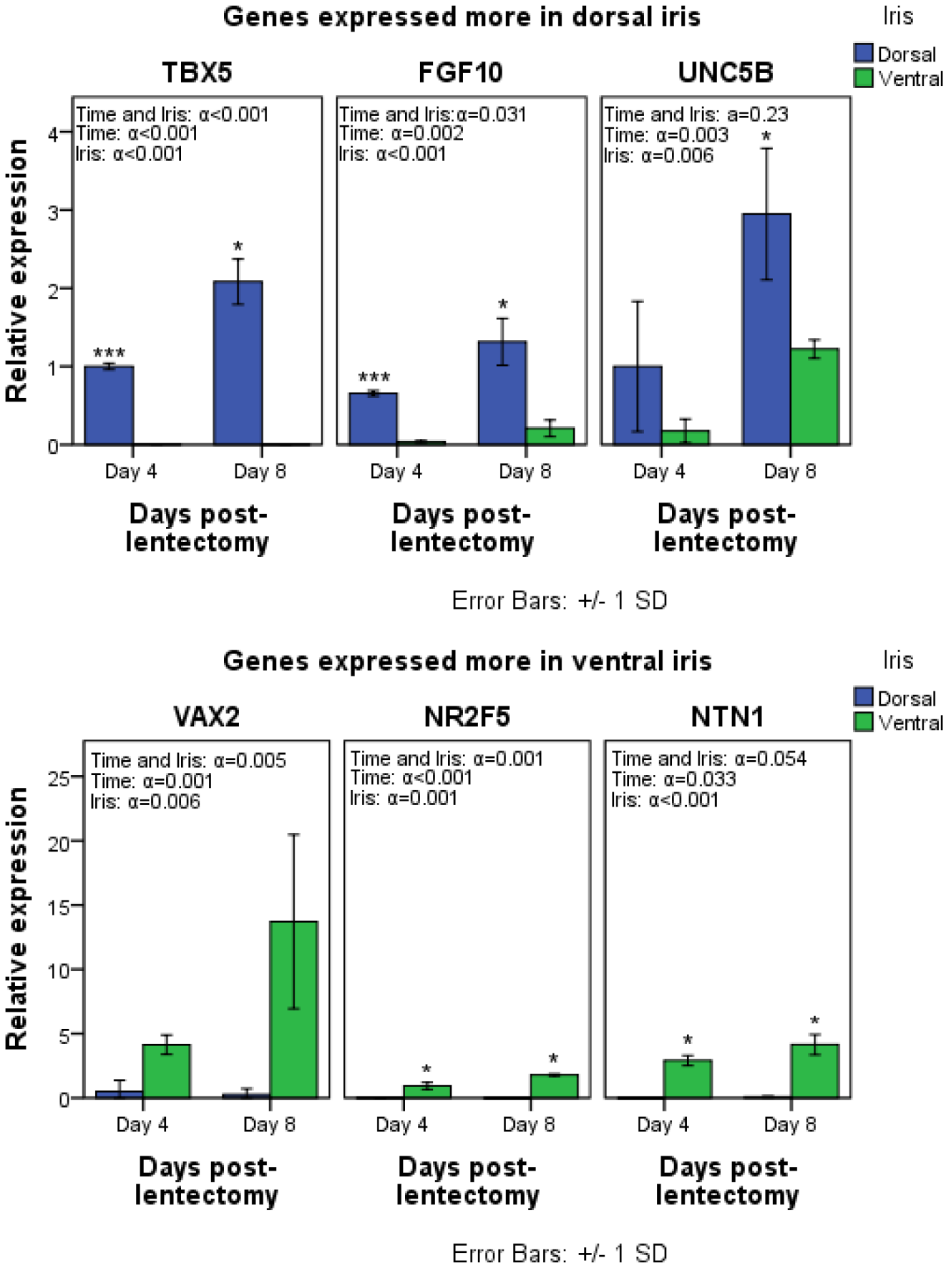
qRT-PCR expression validation of TBX5, FGF10, UNC5B, VAX2, NR2F5 and NTN1. Expression of the different genes at the RNA level is indicated as relative expression. Bars indicate standard deviation. Statistical test was performed with two-way ANOVA and Student’s t-test. Asterisks above the bars indicate statistical significance (*: p<0.05, ***: p<0.001) between dorsal and ventral iris samples of the same day.

We have also verified some of these patterns via qRT-PCR, which confirm this remarkable difference in expression along the dorsal/ventral iris. The up-regulation of TBX5, FGF10 and UNC5B in the dorsal iris and the up-regulation of VAX2, NR2F5 and NTN1 in the ventral iris are shown in Figure 3. Interestingly, the expression of these genes is also dependent on time, suggesting a potential role of those genes during regeneration (ANOVA; α<0.05). In addition, we further verified the up-regulation of NTN1 in the ventral iris and its receptor (UNC5B) in the dorsal iris.

### What Genes are Regulated Specifically at 4 or 8 dpl?

We showed only minor qualitative differences in gene expression between the dorsal and ventral iris. Quantitative changes are dominant and seem to correlate with regenerative abilities. To answer the question whether there are any differences at day 4 (both dorsal and ventral) versus day 8 (dorsal and ventral), which might uncover the importance of timing rather than spatial regulation, we compared the 4 day group with the 8 day group as shown in Figure 4.

Fisher’s exact test with multiple testing corrections for GO from transcripts that are up-regulated in both dorsal and ventral iris 4 dpl compared to 8 dpl versus the whole reference transcripts reveals that DNA polymerase activity and nucleotidyltransferase activity GO terms to be significantly enriched (Table S3). This indicates the initiation of the cell cycle re-entry which has been found to be the major event 4 dpl [149].

Fisher’s exact test with multiple testing corrections for GO from transcripts that are up-regulated in both dorsal and ventral iris 8 dpl compared to 4 dpl versus the whole reference transcripts revealed many interesting patterns (Table S4): as expected GO terms in the cellular component category and related to extracellular matrix are over-represented in the group. Furthermore over-represented terms include extracellular matrix structural constituents and metalloendopeptidase activity in the molecular function category (Table S4). Collagen catabolic process, collagen metabolic process, cell adhesion, extracellular matrix and structure organization, and peptide secretion are over-represented in the biological process category (Table S4). In addition, many of the GO terms in the biological process category related to differentiation, movement, development and patterning are over-represented in the group. Finally, many GO terms that are over-represented in the group are related to macromolecule transport and synthesis. These results indicate active remodeling, transcription and metabolism at day 8, which was expected because the process of dedifferentiation and specification of the lens vesicle peaks at this time point.

### Conclusion

The transcriptome analysis during lens regeneration revealed much needed and useful information. First, we were able to identify quantitative patterns of gene expression that create gradients along the dorsal/ventral iris. This finding is of particular importance since it establishes a molecular framework that drives the ability of the dorsal iris for lens regeneration. Second, our analysis identified genes that might be critical for the induction of lens regeneration. For the first time, we now know factors that can be studied in functional assays, such as in trangenesis or knockdown [150], [151], to establish their potency in inducing/inhibiting regeneration. In addition, this knowledge allows us to perform comprehensive comparisons to other animal models that lack the ability for lens regeneration, which might unveil fundamental differences and similarities between regenerating and non-regenerating species.

## Supporting Information

Table S1 **GO terms that are over-represented in dorsal iris 4 and 8 dpl versus ventral iris 4 and 8 dpl.**
(XLSX)

Table S2 **GO terms that are over-represented in ventral iris 4 and 8 dpl versus dorsal iris 4 and 8 dpl.**
(XLSX)

Table S3 **GO terms that are over-represented in transcripts commonly up-regulated (>2 fold) in dorsal and ventral iris at 4 dpl compared to 8 dpl versus whole reference transcriptome.**
(XLSX)

Table S4 **GO terms that are over-represented in transcripts that are commonly up-regulated (>2 fold) in dorsal and ventral iris 8 dpl compared to 4 dpl versus whole reference transcriptome.**
(XLSX)
